# Prostate derived Ets transcription factor and Carcinoembryonic antigen related cell adhesion molecule 6 constitute a highly active oncogenic axis in breast cancer

**DOI:** 10.18632/oncotarget.934

**Published:** 2013-04-10

**Authors:** Alka Mukhopadhyay, Thaer Khoury, Leighton Stein, Protul Shrikant, Ashwani K Sood

**Affiliations:** ^1^ Department of Immunology, Roswell Park Cancer Institute, Buffalo, NY; ^2^ Department of Pathology, Roswell Park Cancer Institute, Buffalo, NY; ^3^ Department of Gene Expression Core Laboratory, Roswell Park Cancer Institute, Buffalo, NY

**Keywords:** PDEF, SPDEF, CEACAM6, Elevated co-expression, Tumor cell survival, PDEF-CEACAM6 oncogenic axis, Novel targets inbreast cancer

## Abstract

We previously reported overexpression of Prostate derived Ets transcriptionfactor (PDEF) in breast cancer and its role in breast cancer progression, supportingPDEF as an attractive target in this cancer. The goal of this research was to identifyspecific PDEF induced molecules that, like PDEF, show overexpression in breast tumorsand a role in breast tumor progression. PDEF expression was down regulated byshRNA in MCF-7 human breast tumor cell line, and probes from PDEF down-regulatedand control MCF-7 cells were used to screen the HG-U133A human gene chips. Theseanalyses identified 1318 genes that were induced two-fold or higher by PDEF in MCF-7 cells. Further analysis of three of these genes, namely CEACAM6, S100A7 and B7-H4, in relation to PDEF in primary breast tumors showed that in 82% of ER+, 67%of Her2 overexpressing and 24% of triple-negative breast tumors both PDEF andCEACAM6 expression was elevated 10-fold or higher in comparison to normal breasttissue. Overall, 72% (94 of 131) of the primary breast tumors showed 10-fold orhigher expression of both PDEF and CEACAM6. In contrast, S100A7 and B7-H4 failedto show concordant elevated expression with PDEF in primary tumors. To determinethe significance of elevated PDEF and CEACAM6 expression to tumor phenotype, theirexpression was down regulated by specific siRNAs in human breast tumor cell lines. This resulted in the loss of viability of tumor cells in vitro, supporting an oncogenicrole for both PDEF and CEACAM6 in breast cancer. Together, these findings show thatPDEF-CEACAM6 is a highly active oncogenic axis in breast cancer and suggest thattargeting of these molecules should provide novel treatments for most breast cancerpatients.

## INTRODUCTION

Targeted treatments of ER^+^ and Her2 overexpressing breast tumors have significantly improved the clinical outcomes for most breast cancer patients [1, 2]. However, treatment resistance remains the underlying cause of tumor recurrence, metastatic spread and the continuing high mortality rates from this cancer worldwide [3-6]. On the other hand, for the ER^−^, PR^−^ and Her2^−^ (triple-negative) breast tumors that are characterized by generally poor prognosis, few useful targeted treatments are currently available. Evidently, there is a dire need to identify novel oncogenic molecules and/or molecular pathways for developing novel targeted treatments to achieve better tumor control.

PDEF is a relatively novel member of the Ets family of transcription factors that play an important role in the various developmental and cellular processes such as cell lineage specification, proliferation, migration, apoptosis and angiogenesis; and their aberrant expression is a frequent underlying cause of cancer development in humans [7-11]. We and others have previously reported highly restricted expression of PDEF in normal human tissues [12, 13] and its over expression in primary breast tumors [13-15]. Further, elevated PDEF expression also occurs in the precursor lesions including the atypical ductal hyperplasia [16] and in ductal carcinoma in situ [15], and is maintained in lymph node metastases [17]. Moreover, transfection of PDEF into the immortalized MCF-10A and MCF-12A breast epithelial cell lines led to increased clonogenicity *in vitro* and tumorigenicity in immunodeficient mice; and meta-analysis of PDEF expression in relation to clinical outcome showed a significant association of high PDEF expression with poor disease-free and overall survival in independent patient cohorts [16, 18]. These observations established PDEF as a novel oncogene and an attractive target in breast cancer.

Further insights into the identity of the molecules that mediate the oncogenic action of PDEF and may serve as additional targets in breast cancer may be gained from the study of the PDEF induced genes. Accordingly, this communication describes CEACAM6 (carcinoembryonic antigen related cell adhesion molecule 6) as a PDEF induced molecule in breast cancer. CEACAM6 belongs to the human CEA (carcinoembryonic antigen) gene family consisting of seven members within the CEACAM subfamily [19]. Also known as NCA-50/90 or CD66c, CEACAM6 is expressed on the cell surface (anchored *via* the glycophosphotidyl inositol linkage) and is involved in the homophilic and heterophilic interactions in cell adhesion [20, 21]. Deregulated transgenic expression of CEA/CEACAM6 inhibits colonocyte differentiation leading to hyperplasia and dysplasia, implicating a role for this molecule in colon tumor development [22]. Moreover, silencing CEACAM6 by SiRNA enhanced anoikis (apoptosis caused by loss of anchor) and sensitivity to cytotoxic killing of colon and pancreatic tumor cell lines [23, 24]. Since the role of CEACAM6 in human breast cancer and in particular in relation to PDEF remains poorly understood, this communication also describes the characteristics of PDEF and CEACAM6 expression in primary breast tumors and their contributions to the tumor phenotype.

## RESULTS

### Silencing PDEF expression in MCF-7 human breast tumor cell line and identification of PDEF regulated genes

**Figure 1:**
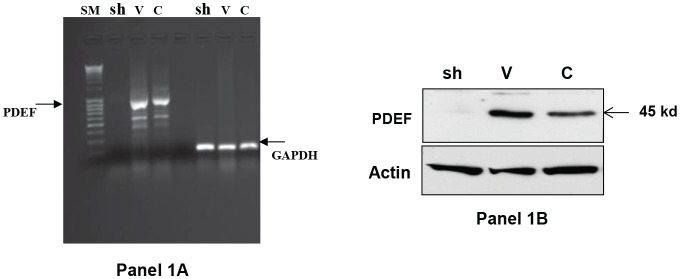
1A Silencing of PDEF expression by small hairpin RNA (shRNA) in MCF-7 breast tumor cell line: The left-most lane in this figure shows the DNA size markers. The next three lanes marked at the top as Sh, V and C respectively show the results of PDEF specific PCR on RNA isolated from MCF-7 cells transfected with PDEF shRNA encoding plasmid, MCF-7 cells transfected with vector plasmid and untransfected control MCF-7 cells. The three right lanes show GAPDH expression in these same cells. Panel 1B shows the western blot data using PDEF specific antibody for the corresponding samples shown in Panel 1A.

PDEF expression was stably down-regulated in MCF-7 breast tumor cell line by transfection with a plasmid (described in Materials and Methods) encoding a PDEF specific shRNA sequence. The down-regulation of PDEF expression was confirmed by RT/PCR and the data are shown in Figure 1, Panel 1A. As shown in lane 2 (labeled as sh) of this panel, PDEF expression was completely abrogated in cells transfected with shRNA plasmid in comparison to vector transfected (lane labeled V) or control un-transfected MCF-7 cells (lane labeled C). The Panel 1B in this figure shows similar loss of PDEF protein expression in the shRNA expressing MCF-7 cells. It is noteworthy that shRNA plasmid-transfected MCF-7 cells formed visible transfectant colonies more than one month post transfection. In contrast, vector plasmid transfected cells formed visible colonies much earlier i.e. at about three weeks post transfection. Apparently, abrogation of PDEF expression by shRNA lead to decreased growth and/or survival of MCF-7 cells. RNA was isolated from PDEF-down-regulated MCF-7 cells and control PDEF-positive MCF-7 cells, labeled and then used to screen the HG-U133A human gene chips from Affymetrix. Two separate experiments were performed and analyzed for changes in gene expression and genes with 2-fold or higher expression in both experiments were considered as PDEF regulated. This analysis identified 1318 genes that were up-regulated 2-fold or higher by PDEF and another 733 genes that were down-regulated 2-fold or higher by PDEF in MCF-7 cells (data available at http://www.ncbi.nlm.nih.gov/geo/query/acc.cgi?acc=GSE37662).

Among the PDEF induced genes 83 showed 5-fold or higher induction by PDEF and they are shown in Supplementary Table 1. Most of these genes have putative roles in cell-cell and cell-matrix adhesion, cell growth/survival, innate or adaptive immunity, bone morphogenesis/growth and in transcription regulation. Of these, three genes including B7-H4 (8.8-fold induction), S100A7 (6.96-fold induction) and CEACAM6 (5.1-fold induction) were of particular interest since they were previously reported to show limited expression in normal human tissues and over expression in breast tumors, hence potentially useful as breast tumor targets in conjunction with PDEF [25-29].

### PDEF and CEACAM6 show elevated co- expression in primary breast tumors

**Figure 2:**
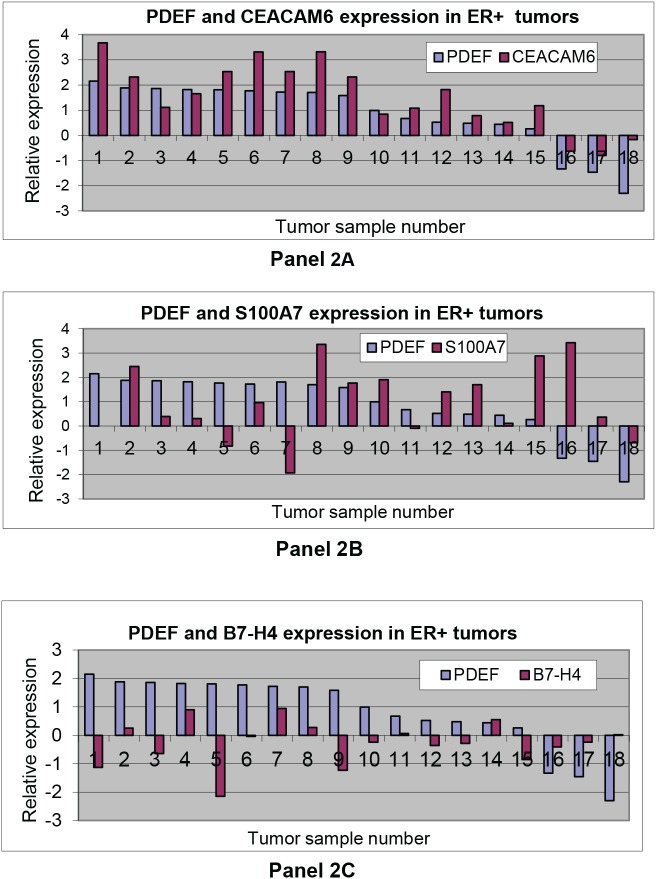
Gene expression analysis of, CEACAM6, S100A7 or B7-H4 in relation to PDEF: Panels 2A, 2B and 2C respectively show CEACAM6, S100A7 or B7-H4 expression in relation to PDEF in 18 tumor samples described in the text qRT-PCR was used to determine expression in tumors and in a mixture of normal breast RNA from 5 healthy women. Relative expression in tumors in comparison to normal breast tissues is shown on a log_10_ scale.

Since we previously reported over expression of PDEF in breast tumors [13, 15] and since PDEF induces the expression of S100A7, CEACAM6 and B7-H4 in MCF-7 cells, we tested whether these molecules show concordant and elevated co-expression with PDEF in primary breast tumors. To that purpose, we first analyzed 93 ER+ breast tumors for PDEF expression by qRT-PCR (data not shown) and selected the top 9 tumors with highest PDEF expression and the bottom 9 tumors with lowest PDEF expression. These 18 tumors were then analyzed for CEACAM6, S100A7 and B7-H4 expression. The data are shown in Figure 2, panels 2A, 2B and 2C respectively for CEACAM6, S100A7 and B7-H4 in relation to PDEF. Thus, for CEACAM6, it was found that 8 of the 9 high PDEF expressing tumors also showed high CEACAM6 expression (tumors #1-9, except tumor #3). Similarly, 8 of the 9 low PDEF expressing tumors showed low levels of CEACAM6 expression (tumors # 10-18, except tumor #12). Together, these results show concordance between PDEF and CEACAM6 expression and elevated co-expression of these molecules in primary breast tumors. In contrast, and as shown in the panels 2B and 2C respectively, S100A7 and B7-H4 failed to show significant concordance and elevated co-expression with PDEF in these tumors.

From Figure 2 panel 2A, it is evident that CEACAM6 is at least 10-fold over expressed even in some of the lowest PDEF expressing tumors, e.g. in tumor numbers 11, 12 and 15. We therefore determined the CEACAM6 expression levels in all 93 tumors and found that 80 of the 93 (86%) tumors over expressed CEACAM6 at 10-fold or higher levels than the normal breast tissue (data not shown). When considering both PDEF and CEACAM6 together, 76 of the 93 (82%) ER+ tumors expressed 10-fold or higher levels of these molecules.

### Correspondence between CEACAM6 mRNA and protein expression in primary tumors

**Figure 3:**
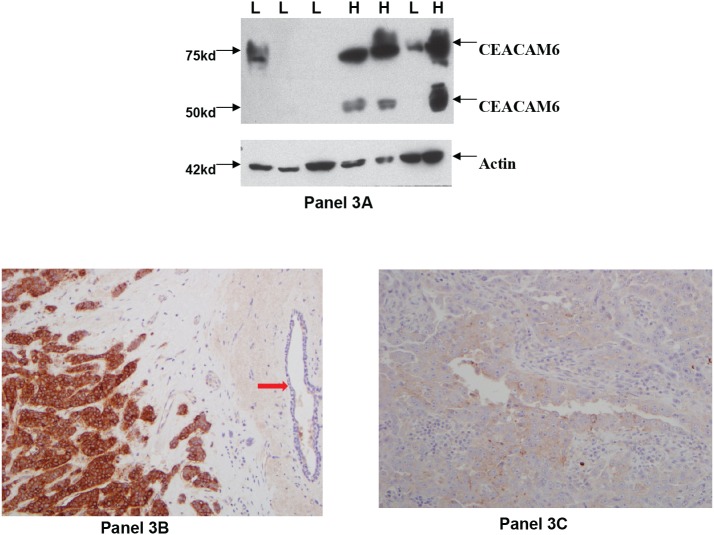
CEACAM6 protein expression in primary breast tumors: CEACAM6 specific antibody 9A6 was used to probe CEACAM6 protein expression by Western blot method The data are shown in Panel 3A. Specifically, the upper part of Panel 3A shows CEACAM6 specific bands corresponding to the two glycosylated (50kd and 75kd) forms. The lower part of Panel 3A shows actin expression in these samples. The letter L denotes low CEACAM6 expressing tumors (CEACAM6-low) and H denotes high CEACAM6 expressing tumors (CEACAM6-high) respectively. The Panels 3B and 3C respectively show immunohistochemical staining of a CEACAM6-high tumor and a CEACAM6-low tumor. The solid pink arrow in panel 3B indicates a normal mammary duct that lacks CEACAM6 expression.

To determine whether CEACAM6 mRNA and protein levels correspond, we tested CEACAM6 protein expression by Western blotting. Of the 18 tumor samples shown in Figure 2, frozen tissue was available for protein isolation and Western blot analysis for 7 tumors. These included three CEACAM6-high and four CEACAM6-low mRNA expressing tumors. The data for CEACAM6 protein expression by Western blot are shown in Figure 3, panel 3A. The results show a correspondence between CEACAM6 mRNA and protein levels in these tumors, suggesting that, like CEACAM6 mRNA, CEACAM6 protein is similarly elevated in primary breast tumors.

To gain further insights into the nature of CEACAM6 protein expression in primary tumors, we analyzed the formalin fixed paraffin embedded (FFPE) tissue sections from these tumors by immunohistochemical staining for CEACAM6 expression. The representative results are shown in Figure 3, panels 3B for a CEACAM6-high tumor and in panel 3C for a CEACAM6-low tumor. As seen in Panel 3B, strong cell surface expression of CEACAM6 was observed in CEACAM6-high tumor. The solid pink arrow in Panel 3B indicates a normal mammary duct present within this section that does not detectably stain with the antibody, confirming over expression in tumor cells in comparison to benign epithelial cells.

### Elevated co-expression of PDEF and CEACAM6 in Her2^+^ and triple-negative breast tumors

**Figure 4:**
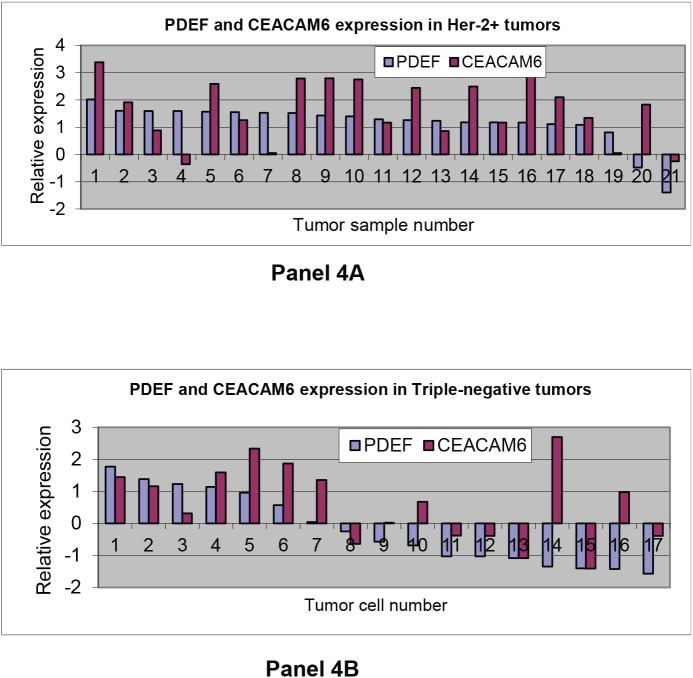
Gene expression analysis of CEACAM6 and PDEF in Her-2 over expressing and in triple-negative breast tumors: The panels 4A and 4B respectively show CEACAM6 expression in relation to PDEF in 21 Her-2 over expressing tumors and 17 triple-negative tumors The qRT-PCR method was used to determine the expression in tumors and in a mixture of normal breast RNA from 5 healthy women. Relative expression in tumors in comparison to normal breast tissues is shown on a log_10_ scale.

The highly frequent over expression of PDEF and CEACAM6 in ER+ breast tumors prompted us to examine whether similar elevated co-expression of these molecules also occurs in Her2 over expressing (Her2^+^) and triple-negative breast tumors. Accordingly, PDEF and CEACAM6 expression was analyzed by qRT-PCR in 21 Her2^+^ tumors and in 17 triple-negative breast tumors. The results are shown in Figure 4, panels 4A for Her2^+^ tumors and in panel 4B for triple-negative breast tumors respectively. Specifically, 14 of the 21 (67%) Her2^+^ tumors showed 10-fold or higher expression of both PDEF and CEACAM6. On the other hand, in the triple-negative breast tumors 4 of 17 (24%) showed 10-fold or higher expression of both PDEF and CEACAM6. If considering PDEF and CEACAM6 separately, for Her2^+^ tumors 18 of 21 (86%) showed 10-fold or higher expression of PDEF and 15 of 21 (71%) showed 10-fold or higher expression of CEACAM6 (Figure 4, panel 4A). For triple-negative tumors, the frequency of over expression for PDEF remained relatively low, i.e. 5 of 17 (29%) tumors showing 10-fold or higher expression. In contrast for CEACAM6, 8 of 17 (47%) triple-negative tumors showed 10-fold or higher expression of this molecule (Figure 4, panel 4B). Overall, these results showed elevated co-expression of PDEF and CEACAM6 in 67% of Her2^+^ breast tumors, but significantly less frequent (24%) elevated co-expression of these molecules in triple-negative tumors.

### Down regulation of PDEF and CEACAM6 expression by specific siRNAs reduces the viability of cells from BT-474 and SKBR3 human breast tumor cell lines

**Figure 5:**
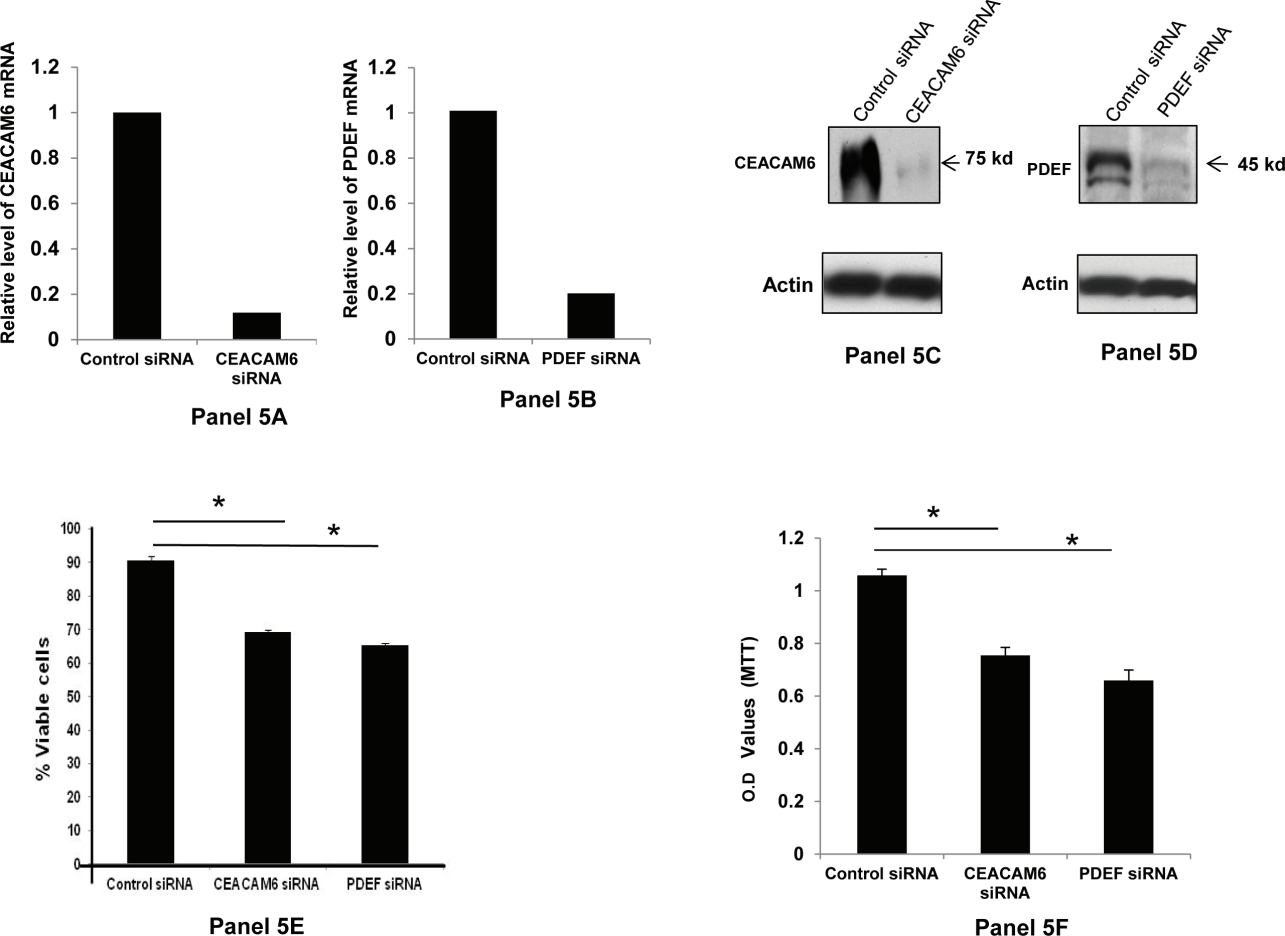
Down regulation of CEACAM6 and PDEF expression and its effect on BT-474 cell survival: Panels 5A and 5B respectively show the relative down regulation of CEACAM6 and PDEF mRNAs following treatment with specific siRNAs, as measured by qRT-PCR The panels 5C and 5D shows the corresponding changes in the CEACAM6 and PDEF protein expression as determined by Western blotting with specific antibodies. Panel 5E shows the percent viable cells in the specific siRNA-treated BT-474 cells relative to those in the control siRNA treated BT-474 cells. Similarly, the Panel 5F shows the relative loss of cell viability in the specific siRNA treated BT-474 cells compared to control siRNA treated cells, as measured by the MTT assay. The O.D. values in Panel 5F are a measure of the metabolically active cells present in the experimental and control samples. The asteriscs (*) in panels 5E and 5F denote the significant p value of <0.01.

**Figure 6:**
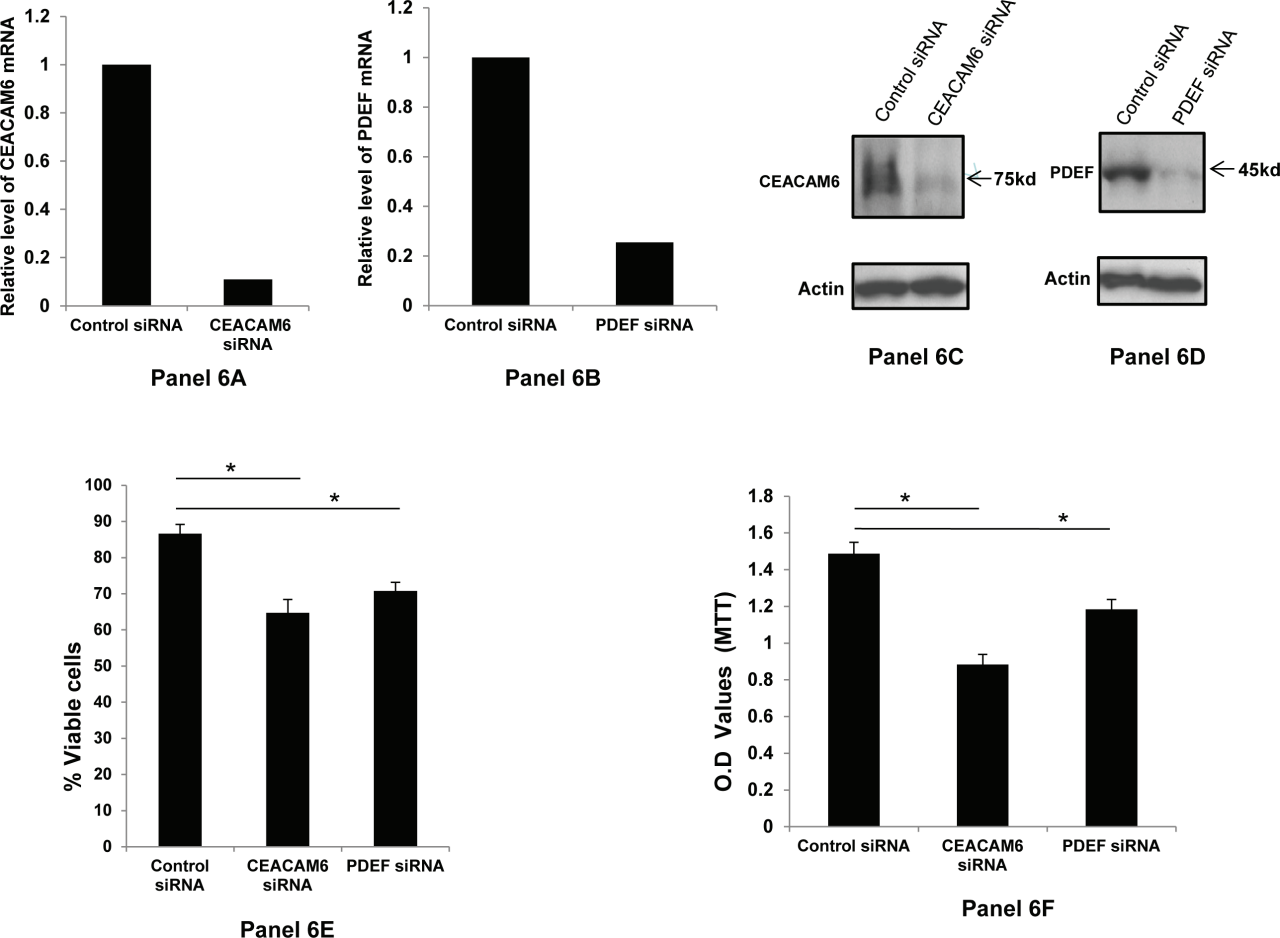
Down regulation of CEACAM6 and PDEF expression and its effect on SKBR3 cell survival: Panels 6A and 6B respectively show the relative down regulation of CEACAM6 and PDEF mRNAs following treatment with specific siRNAs, as measured by qRT-PCR The panels 6C and 6D respectively shows the corresponding changes in the CEACAM6 and PDEF protein expression as determined by Western blotting with specific antibodies. Panel 6E in this figure shows the percent viable cells in the specific siRNA-treated SKBR3 cells relative to those in the control siRNA treated cells. Similarly the Panel 6F shows the relative loss of cell viability in the specific siRNA treated SKBR3 cells compared to control siRNA treated cells, as measured by the MTT assay. The asterics (*) in panels 6E and 6F denote significant p value of <0.01.

To determine the contribution of the elevated expression of PDEF and CEACAM6 to the tumor phenotype, their expression was down regulated by specific siRNA in BT-474 and SKBR3 human breast tumor cell lines. The cell viability was determined by the trypan blue dye exclusion assay and by the MTT assay. The results of these experiments are shown in Figure 5 for BT474 cells and in Figure 6 for SKBR3 cells. Briefly for BT474 cells, the panels 5A and 5B show the representative quantitative PCR (qRT-PCR) data on the relative down regulation of CEACAM6 and PDEF by the respective siRNA treatment. As shown, the CEACAM6 mRNA expression was down regulated by CEACAM6 specific siRNAs to 11% of the value for control siRNA treated BT-474 cells (Panel 5A). Similarly, PDEF mRNA expression was down regulated by PDEF specific siRNAs to 19% of the value of the control siRNA treated BT-474 cells (Panel 5B). The corresponding protein level down regulation for these molecules is shown in panels 5C and 5D, and these results were consistent with those shown in panels 5A and 5B. The data in panels 5E and 5F show that CEACAM6 and PDEF down regulation significantly reduced the viability of BT-474 cells as determined by both trypan blue dye exclusion assay (Panel 5E) and by the MTT assay (Figure 5F). Specifically, the viable cell count by trypan blue dye exclusion assay was reduced from 90% for control siRNA treated cells to 69% (p<0.01) and 65% (p<0.01) respectively for CEACAM6 siRNA and PDEF siRNA treated cells. Similarly, the cell viability determination by MTT assay showed significant loss of viability of BT-474 cells following the CEACAM6 (p<0.01) or PDEF (p<0.01) specific siRNA treatment compared to control siRNA treated cells.

Similar to BT-474 cells, the data in Figure 6 show that significant loss of cell viability also occurs when SKBR3 cells are treated with CEACAM6 or with PDEF specific siRNAs. Specifically, the loss of viability following CEACAM6 down regulation is similar for both BT-474 and SKBR3 cells and this is consistent with similar level of down regulation of CEACAM6 in these two cell lines (compare Figure 5, Panel 5A and Figure 6, Panel 6A). However, the loss of viability is slightly lower for SKBR3 compared BT-474 cells following the down regulation of PDEF. This may be attributed to the relatively lower level of PDEF down regulation in SKBR3 cells i.e. to 25% level relative to control siRNA (Panel 6B) in comparison to BT474 cells i.e. to 19% level relative to control siRNA (Panel 5B). Nevertheless, the results on the loss of tumor cell viability shown in Figure 6 are reproducible in separate experiments (n=3) and are significant (p=<0.01) for both CEACAM6 and PDEF and by both trypan blue dye exclusion assay (Panel 6E) and by MTT assay (Panel 6F).

## DISCUSSION

A novel finding of this study is that CEACAM6 is a PDEF induced gene. This association between PDEF and CEACAM6 was not reported previously in the gene expression profiling analysis of PDEF regulated genes [30]. This may be due to the use of different breast tumor cell lines in the two studies. Specifically, we down-regulated the expression of PDEF in the ER^+^ MCF-7 breast tumor cell line of luminal epithelial origin. In contrast, Turner *et al* transfected the ER-negative breast tumor cell lines of the basal epithelial origin [30]. Presumably, the availability of specific co-activators/transcription regulators in the luminal versus basal epithelial breast tumor cell lines may differ and lead to the differences in the regulation of CEACAM6 by PDEF. The largely concordant expression of PDEF and CEACAM6 in primary breast tumors (Figure 2) is also consistent with the notion that CEACAM6 is a PDEF induced gene in breast cancer.

Our screening of the HG-U133A human gene chips with probes from the PDEF-expressing MCF-7 cells and PDEF-lacking MCF-7 cells showed that PDEF regulates the expression of a large number of genes in the MCF-7 breast tumor cell line. However, further analysis of three of the PDEF induced genes, namely S100A7, CEACAM6 and B7-H4 in primary human breast tumors revealed that only CEACAM6 showed concordance and elevated co-expression with PDEF in primary tumors. The lack of concordance between PDEF and S100A7 or PDEF and B7-H4 expression in primary tumors underscores the need to confirm the cell line derived data in primary tumors, in order to determine the potential clinical significance of the specific gene expression changes observed in the gene-chip microarray screens of breast tumor cell lines. Presumably, in primary tumors, other factors of tumor cell origin and/or those contributed by the stromal cells present in the tumor microenvironment may alter the expression of S100A7 and B7-H4, thereby masking their PDEF mediated induction. Alternatively these genes might not be the direct transcriptional targets of PDEF, but rather indirect consequence of other PDEF regulated genes in the stably transfected MCF-7 cells.

CEACAM6 was previously reported to be overexpressed in breast cancer however, the level of CEACAM6 over expression appeared modest presumably since immunohistochemistry alone was used to assess CEACAM6 expression [28]. In contrast, our analysis used the qRT-PCR, Western blotting and immunohistochemistry methods and found highly elevated expression of CEACAM6 in most breast tumors. This may be appreciated from the data shown in Figure 2, panel 2A and in Figures 3 and 4. Specifically, the level of over expression in the 8 CEACAM6 high tumors shown in Figure 2, panel 2A ranged from 46-fold for tumor #4 to 4705-fold for tumor #1). Moreover, from the analysis of 131 primary tumors, 86% of ER+, 71% of Her2+ and 47% of triple negative breast tumors exhibited 10-fold or higher levels of CEACAM6 expression. This study therefore provides a more detailed understanding of the CEACAM6 expression in breast cancer.

Perhaps the most important findings of this study are the elevated co-expression of PDEF and CEACAM6 in a high percentage (72%) of the primary human breast tumors (Figures 2A, 3 and 4) and the essential role of these molecules in the survival of the human breast tumor cell lines (Figure 5 and 6). These results confirm the oncogenic role of PDEF in breast cancer; however, to our knowledge a causative role of CEACAM6 in breast tumor growth and/or survival was not reported previously. Hence, our findings that down regulation of CEACAM6 by siRNA led to the loss of the viability of BT474 and SKBR3 cells (Figures 5 and 6) are novel and demonstrate an oncogenic role for CEACAM6 in breast cancer. These results together with the frequent elevated co-expression of these molecules establish PDEF-CEACAM6 as a highly active oncogenic axis in breast cancer. Moreover this oncogenic axis is active across the different breast tumor types characterized by the hormone receptor and Her2 expression status. This widespread elevated co-expression across the breast tumor types is perhaps why this oncogenic axis was not discovered previously in the microarray-based expression profiling studies of primary breast tumors. Presumably, in those studies the main focus was to identify the gene expression signatures specific to the individual tumor subtypes [31].

The results presented in this study also suggest high promise of targeting PDEF and CEACAM6 in breast cancer, independent of and/or in conjunction with the existing ER and Her2 targeted treatments of hormone receptor-positive and Her2 over expressing tumors. The elevated co-expression in 24% of triple negative breast tumors further suggests the potential for developing novel targeted approaches to the treatment of these tumors for which targeted treatments are not currently available. The importance of PDEF and CEACAM6 as targets is further evident when considering the results from the comprehensive genome wide sequencing and expression analysis of a large number of human breast tumors [reviewed in 32 and 33]. Specifically, significant novel targetable oncogenes were not identified in these studies [32]. Moreover, with the exception of the PI3K oncogene that suffered mutations in 30-35% of breast tumors, the vast majority of the frequently occurring mutations in these tumors were restricted to tumor suppressor genes that are difficult to target [32, 33].

How PDEF and CEACAM6 over expression may promote breast tumor progression may be envisioned as follows. Based on the data from Figures 5 and 6, one mechanism appears to be to promote enhanced survival of tumor cells. Secondly, the elevated CEACAM6 expression on tumor cells may increase intercellular interactions between tumor cells (since CEACAM6 is a cell adhesion molecule), leading to their increased adhesion and the formation of cellular aggregates. These tumor cell aggregates may exhibit increased arrest in the capillary beds during circulation resulting in increased metastatic efficiency. Moreover, among the non-epithelial tissues granulocytes and myeloid cells express CEACAM6 at appreciable levels [29]. This further suggests the potential to form mixed tumor cell-myeloid cell aggregates that in addition to contributing to increased metastatic efficiency may also induce immune suppression in the tumor microenvironment. Further research is needed to test these hypotheses and to develop a better understanding of the role of the PDEF-CEACAM6 oncogenic axis in breast cancer.

## MATERIALS AND METHODS

### Cell lines and reagents

MCF-7, BT-474 and SKBR3 human breast tumor cell lines were purchased from American Type Culture Collection. The authenticity of these cell lines is periodically verified by the supplier by short tandom repeat DNA profiling and by specific isoenzyme expression. These cell lines were grown in Dulbecco's Modified Eagle Medium supplemented with 10% fetal bovine serum. Mouse anti-CEACAM6 monoclonal antibody 9A6 (GM0509) was purchased from Aldevron, USA and mouse anti-actin antibody was purchased from Sigma, USA. Anti-PDEF rabbit polyclonal antibody was prepared in our lab as described previously (15). PDEF, CEACAM6, S100A7, B7-H4 and GAPDH specific TaqMan gene expression assays and the associated reagents were purchased from Applied Biosystems Inc, USA.

### shRNA mediated down-regulation of PDEF expression

Using optimal criteria in an algorithmic program from InvivoGen, USA, a 21 nucleotide sense PDEF sequence (shown underlined) and its complement (shown in italics and underlined) from the coding region of the PDEF mRNA were selected to design the PDEF shRNA encoding DNA sequence:5'--TCCCACCTGGACATCTGGAAGTCAGTCAAGAG*CTGACTTCCAGATGTCCAGGT*TT-3' (sense)5'-CAAAAAACCTGGACATCTGGAAGTCAGCTCTTGA*CTGACTTCCAGATGTCCAGGT*-3' (anti-sense). These two DNA sequences were annealed and cloned into psiRNAH1zeo vector (InvivoGen, USA) at the BbsI site to generate plasmid pPDEF-shRNA expression vector. After sequence confirmation, 5 μg each of the pPDEF-shRNA plasmid or vector plasmid were transfected into MCF-7 cells in serum-free medium using lipofectamine^TM^2000 Plus and following the manufacturer's instructions (Invitrogen, USA). Stable transfectants were selected using Zeocin (500 μg/ml). After drug selection, transfectants were evaluated for PDEF expression by RT-PCR and Western blotting.

### Analysis of PDEF expression in MCF-7 transfectants

RNA was isolated from PDEF shRNA plasmid transfected, vector plasmid transfected and control untransfected MCF-7 cells by Tri reagent^TM^ (Molecular Research Center Inc Cincinnati, OH) using manufacturer's instructions and tested for quality on 1% agarose/formaldehyde gel. Intact 28S and 18S ribosomal RNA bands were used as criteria for RNA quality for further use. The expression of PDEF and GAPDH was analyzed by RT-PCR using the following primers. For PDEF, 5'-ATGGGCAGCGCCAGCCCGGGTC-3^'^ (sense) and 5'-TCAGATGGGGTGCACGAACTGGT-3' (anti-sense); and for GAPDH, 5^'^-GCTTCCCGTTCTCAGCCTTGAC-3' (sense) and 5^'^-ATGGGAAGGTGAAGGTCGGAG-3^'^ (anti-sense) primers were used. For PDEF protein expression analysis, cell lysates were prepared and analyzed by Western blotting using anti-PDEF antibody.

### Screening HG-U133A human gene chips and identification of PDEF regulated genes

Hybridization probes were generated from RNA isolated from PDEF shRNA plasmid transfected and control vector plasmid transfected MCF-7 cells by using the Affymetrix protocol. Briefly, cells were washed twice in PBS and then homogenized in Trizol using a Polytron® homogenizer. Samples were prepared for GeneChip analysis as described in the Affymetrix GeneChip Expression Analysis Manual (Affymetrix Inc, Santa Clara, CA). Total RNA was isolated and cleaned using RNeasy columns and the quality assessed using the Agilent Bioanalyzer Lab on Chip. Using 40μg of total RNA double stranded cDNA was synthesized using the Superscript Choice System. A T-7(dT24) primer was used to prime the first strand cDNA synthesis. An in-vitro transcription reaction, which amplifies and biotinylates the samples, was then performed. For this step a BioArray® was used and the template labeled by incorporation of biotinylated-11-CTP and 16-UTP ribonucleotides (ENZO Diagnostics, USA). At each stage of this process the quality of the samples was monitored using both gel electrophoresis and spectrophotometry readings. The biotinylated cRNA was then purified, fragmented and hybridized to Affymetrix HG-U133A GeneChip arrays and scanned following streptavidin-phycoerythrin staining. Partek Genomics Suite Software was used to identify transcripts that were up or down regulated 2-fold or greater in the control (PDEF+) versus shRNA transfected (PDEF-) MCF-7 cells. These data were submitted to Gene Expression Omnibus database and can be accessed at http://www.ncbi.nlm.nih.gov/geo/query/acc.cgi?acc=GSE37662).

### Tissue procurement and RNA isolation from primary breast tumors

All patients (n=131) included in this study had a diagnosis of invasive ductal carcinoma of the breast. The tumors included the three major breast tumor types characterized by the hormone receptor and Her2 status and constituted 93 ER^+^, 21 Her2 over expressing (Her2^+^) and 17 triple-negative tumors as determined by immunohistochemistry. The Roswell Park Cancer Institute Institutional Review Board approved this study. Total RNA was isolated from breast tumor samples using the Trizol Reagent (Ambion, Life Technologies Inc, USA) according to manufacturer's instruction. The quality of the isolated RNA was checked by RIN (RNA integrity number) using the Agilent Bioanalyzer and RIN value was from 7 to 9. Total RNA mixture from normal breast tissue from 5 healthy women was purchased from BioChain Institute, Inc, USA.

### Gene Expression by qRT-PCR

For quantitative RT-PCR, 1μg of the total RNA from each breast tumor as well as normal breast RNA was reverse transcribed using the High-Capacity cDNA Reverse Trancription Kit (cat# 4368814, Applied Biosystems, Inc, USA). The manufacturer's instructions were followed, using the Random Primers and MultiScribe™ Reverse Transcriptase enzyme. The reactions were programmed for 10 min at 25°C followed by 120 min at 37°C in a Peltier Thermalcycler PCR machine (MJ research Inc, USA). The cDNA synthesized was then used as template in the qPCR experiments using the specific gene expression probes. Real time amplification was performed on a 7900HT Fast system with standard 96-well block (Applied Biosystems, Inc, USA) using the TaqMan Gene expression Master Mix (cat# 4369016, ABI). The reactions were set in triplicate for each sample in a 10μl volume comprising of cDNA template with the specific TaqMan Gene Expression assay for PDEF, CEACAM6, S100A7, B7-H4 or GAPDH. The endogenous control GAPDH expression level was used in calculating the ΔCt values for specific genes in tumor and normal breast samples. Relative expression in tumor versus the normal breast tissue was calculated by formula 2^−ΔΔCt^, where-in ΔΔCt represents the difference in the ΔCt values of a specific gene in the tumor versus normal breast tissue as control.

### Western Blot Analysis

Frozen Breast Tumor tissue specimens from 7 patients were used for the preparation of the protein lysates. About 0.1g tumor tissue was homogenized in 0.5ml of ice cold RIPA buffer (150mM NaCl, 1% NP-40, 0.5% Sodium Deoxycholate, 0.1%SDS and 50mM Tris, pH8.0) containing protease inhibitor cocktail (Roche). Subsequently the lysates were briefly sonicated (few cycles of 15 sec) and clarified by centrifugation at 12000 rpm, 4°C. The total protein concentration was measured using the Pierce BCA protein assay kit (Thermo Scientific Inc, USA) with BSA as standard and according to the manufacturer's instructions. 50μg protein lysate from each tumor tissue were subjected to 10% SDS-PAGE, and the resolved proteins were electroblotted onto nitrocellulose membrane (Bio-Rad). After blocking 1hr at room temp in 5% non fat milk powder, membranes were incubated with 4ug/ml anti human CEACAM6 monoclonal antibody 9A6 (Aldevron, USA) and anti-actin rabbit polyclonal antibody (Sigma) at 4°C overnight with shaking. Subsequently membranes were washed in TBST buffer and incubated with HRP conjugated goat anti mouse /rabbit antibodies (Bio-Rad). The protein bands were visualized with SuperSignal West Pico enhanced chemiluminescence substrate (Thermo Scientific Inc, USA) using Kodak Film.

### Immunohistochemical analysis of CEACAM6 expression

Paraffin embedded tumor tissue sections were cut at 4 micrometer, placed on charged slides, and dried at 60°C for one hour. Slides were cooled to room temperature, deparaffinized in three changes of xylene, and rehydrated using graded alcohols. For antigen retrieval, slides were treated by heating in the microwave for 10 minutes in citrate buffer (pH 6.0), followed by a 15 minute cool down. Endogenous peroxidase was quenched with aqueous 3% H_2_O_2_ for 10 minutes and washed with PBS/T. Slides were loaded on a DAKO autostainer and serum free protein block (DAKO catalog #X0909) was applied for 5 minutes, blown off, and the anti-CEACAM6 antibody was applied for one hour. Positive and negative control slides were supplied by the pathology core. Labeled polymer HRP anti-Mouse (DAKO # K4007) Envision was then applied, followed by the DAB chromagen (DAKO). Finally, the slides were counterstained with hematoxylin, dehydrated, cleared and cover slipped.

### siRNA Transfection to down regulate CEACAM6 and PDEF expression

Mixtures of two “Silencer Select” siRNAs (Invitrogen) each for CEACAM6 and PDEF were used to down regulate their expression. Their sequences are shown below.

For CEACAM6
Sense (5^′^-3^′^) GCCCUGGUGUAUUUUCGAUtt
Antisense (5^′^-3^′^) AUCGAAAAUACACCAGGGCtg
And
Sense (5^′^-3^′^) CCGGACAGUUCCAUGUAUAtt
Antisense (5^′^-3^′^) UAUACAUGGAACUGUCCGGtt
For PDEF
Sense (5^′^-3^′^) CCUGGACAUCUGGAAGUCAtt
Antisense (5^′^-3^′^) UGACUUCCAGAUGUCCAGGtg
And
Sense (5^′^-3^′^) CUGUCCGCCUUCUACCUCUtt
Antisense (5^′^-3^′^) AGAGGUAGAAGGCGGACAGtg

Briefly, BT-474 or SKBR3 cells were seeded at 2-4×10^5^ cells (30-50% confluency) per well of a 6-well culture dish before the day of transfection. Next day, transfection complexes were made in OPTI-MEM serum-free media (Invitrogen) comprising of 100 nM of the siRNA pools with gentle mixing of the INTERFERin transfection reagent (cat#409-10, Polyplus Transfection, France) according to the manufacturer's instructions. The siRNA and transfection reagent complexes were incubated for 10 min at room temperature and then added to the adherent cells in a final volume of 2.2 ml complete media per well of the 6-well culture dish. After 48 hr incubation the transfected cells were harvested and used for protein and RNA expression analysis and for cell viability assays.

### Cell viablility assays

The siRNA transfected BT-474 or SKBR3 cells at 48hr following transfection were counted for viable cell number by the Trypan blue dye exclusion assay and percent viable cells were calculated. Also the MTT cell viability assay was used according to the standard procedures with minor modifications (Invitrogen protocols). This assay is based on the conversion of the tetrazolium salt MTT (3-(4, 5-dimethythiazol-2-yl)-2, 5-diphenyl tetrazolium bromide) to formazan crystals by metabolically active cells. Briefly, at 48 hr following transfection with siRNA, the transfected cells were seeded at 30,000 cells for BT-474 and 10,000 cells/ well for SKBR3 in triplicate for each sample in a 96 well plate. Next day, medium was changed with 100ul serum free media and the MTT solution was added (10ul of stock solution, 5mg/ml) and the cells were incubated for 4 hours. At the end of the incubation, media was removed and solubilizing agent DMSO was added to each well to dissolve the Formazan crystals formed. After gentle mixing the absorbance of the purple color formed was measured at 540nm in a spectrophotometer. The significance of the differences in cell viability between the mean values within groups was tested by using Student's *t* test. The data are presented as mean from 3 separate experiments plus standard deviation. Differences were considered to be statistically significant at *P<*0.05.

## Supplementary Tables


