# Supplementary material for: Prostate derived Ets transcription factor and Carcinoembryonic antigen related cell adhesion molecule 6 constitute a highly active oncogenic axis in breast cancer
Source: Oncotarget. 2013 Apr 10;4(4):610–21. doi: 10.18632/oncotarget.934 (PMC3720608; doi:10.18632/oncotarget.934)
Supplement: Supplementary file 1 [file oncotarget-04-610-s001.docx]

**Supplementary Table 1. Genes up regulated 5-fold or higher by PDEF in MCF-7 breast tumor cell line**

| Gene Name | Fold Change | Function/role in cancer |
| --- | --- | --- |
| Corneodesmosin | 19.70 | Cell adhesion |
| Mucin 5B, oligomeric mucus/gel forming | 13.93 | Extracellular matrix component; supports innate immunity |
| Chloride channel accessory 2 | 13.00 | Trans epithelial ion transport and protein secretion |
| Proxisome proliferator-activated receptor gamma, cofactor 1 alpha | 13.00 | Transcription co-regulator; inhibits FOXO tumor suppressor; Cell growth/survival |
| Stearoyl-CoA desaturase (delta-9-desaturase) | 11.71 | Fatty acid metabolism; converts long chain fatty acids to mono unsaturated fatty acids; siRNA treatment inhibits proliferation and induces apoptosis in tumor cells; Cell growth/survival |
| DnaJ (Hsp40) homolog, subfamily C, member 6 | 11.71 | Unknown |
| STEAP family member 4 | 10.93 | Involved in secretory/endocytic pathway; over expression induces cell growth and colony formation in prostate tumor cells; Cell growth/survival |
| Matrix Gla Protein | 10.56 | Bone formation; associates with increased migration of glioma tumor cells. |
| Acyl-CoA synthase bubblegum family member 1 | 10.56 | Fatty acid metabolism; cell growth/survival |
| HEG homolog 1 (zebrafish) | 10.20 | Promotes blood vessel formation; over expression may promote angiogenesis; Cell growth/survival |
| Mucin 5AC, oligomeric mucus/ gel forming | 10.20 | Extracellular matrix component; supports innate immunity; promotes cell-matrix adhesion and invasion |
| Spectrin beta, non-erythrocytic 1 | 9.85 | Binds actin; cell-skeletal organization; modulates TGF-beta signaling via regulating nuclear location of smad3 and smad4 proteins |
| Solute carrier family 11, member 1 | 9.85 | Controls susceptibility to several infections; expressed by macrophages; supports innate immunity |
| Catenin, alpha 2 | 9.51 | Controls stability of dendritic spines and of cell adhesion in neurons |
| Desmocollin 2 | 9.19 | Contribute to cell adhesion at desmosomes |
| RCAN family member 3 | 8.88 | Binds calcineurin and inhibits calcineurin dependent activation of NFAT transcription factors in T cells and this inhibits cytokine secretion by T cells. Role in cancer not known. |
| Plaminogen activator, tissue | 8.88 | Reduced migration in neurons in PLAT -/- mice |
| Alanine-glyoxalate aminotransferase | 8.88 | Glyoxalate metabolism |
| V-set domain containing T cell activation inhibitor 1 | 8.88 | Negative regulation of T cell immunity |
| Immunoglubulin heavy chain constant alpha 1 and gamma 1 | 8.57 | Inhibits antibody dependent cellular cytotoxicity |
| CDV3 homolog (mouse) | 8.57 | Unknown |
| Gamma-aminobutyric acid (GABA) A receptor, gamma 2 | 8.28 | Neurotransmitter receptor; role in cancer not known |
| ATP-binding cassette, subfamily A (ABC1) member 8 | 8.00 | Transmembrane transporter, ATP-dependent |
| Tumor necrosis factor receptor superfamily, member 11B | 8.00 | Supports bone growth; Decoy receptor for RANKL, inhibits osteoclast differentiation; cell growth/survival by inhibiting TRAIL activity. |
| UDP glucuronosyltransferase 2 family, polypeptide A3 | 7.73 | Inactivates steroid hormones and their metabolites by glucuronidation |
| Integrin-binding sialoprotein (bone sialo protein) | 7.21 | Bone formation; binds alpha (V)beta3 integrin and promotes cell adhesion to bone matrix; elevated expression associates with bone metastasis of breast and prostate cancers. |
| Zinc finger protein 780B | 7.21 | DNA binding; regulation of transcription |
| Ceroid-lipofuscinosis, neuronal 8 | 7.21 | Glycolipid metabolism |
| Leucine rich repeat containing 31 | 7.21 | Unknown |
| Wiskott-Aldrich syndrome-like | 6.96 | Activated by estradiol; promotes tumor cell migration |
| S100 calcium binding protein A7 | 6.96 | Pro-inflammatory protein; supports innate immunity |
| Amylase, alpha 1A(salivary) | 6.96 | Hydrolyzes oligo and polysaccharides |
| Tripartite motif-containing 29 | 6.73 | Transcription regulation; inhibits p53 and suppresses cell apoptosis; cell growth/survival |
| Protocadherin gamma subfamily A, 10 | 6.73 | Cell adhesion; neuron survival |
| Fucosyl transferase 3 | 6.73 | Cell adhesion to E-selectin and facilitates tumor cell metastasis |
| Parathyroid hormone | 6.50 | Bone formation inducer |
| Deoxyribonuclease I-like2 | 6.50 | DNA degradation in hair and nail corneocytes; part of programmed cell death |
| Nuclear transcription factor Y, alpha | 6.50 | Transcription factor; Positively regulates transcription from RNA Pol II promoters |
| KIAA1109 | 6.28 | Unknown |
| Adaptor-related protein complex 4 Epsilon-1 | 6.28 | Unknown |
| Disabled homolog 2 | 6.28 | Inhibits Wnt signaling; presumably supports epithelial organization |
| Bicaudal C homolog 1 (Drosophila) | 6.28 | Supports cadherine mediated cell adhesion; loss induces Wnt signaling |
| Inhibitor of Bruton agammaglobulinimia tyrosine kinase | 6.28 | Attenuates antibody response by binding Burton tyrosine kinase. Elevated expression may inhibit antibody mediated adaptive immunity |
| Palladin, cytoskeletal associated | 6.06 | Component of cytoskeleton; overexpression induces podosome formation; may promote cell detachment/migration. |
| EPH receptor A4 | 6.06 | Protein tyrosine kinase activity; cell growth/survival; Elevated expression associates with poor clinical outcome in breast cancer |
| Usher syndrome 2A | 6.06 | Binds fibronectin and collagen IV for stable integration into basement membrane; Cell-matrix adhesion |
| Ataxin 7-like 3B | 6.06 | Unknown |
| Basic helix-loop-helix family, member 41 | 6.06 | Transcription factor; siRNA treatment induces apoptosis in paclitaxel-treated MCF-7 cells; cell growth/survival |
| Prolactin receptor | 6.06 | Receptor protein tyrosine kinase, mammary gland development; increased expression induces transformation of mammary epithelial cells; cell growth/survival |
| SMG-7 homolog, nonsense mediated | 5.86 | Functions in nonsense mediated RNA decay (NMD) to preserves cell integrity |
| Nidogen 2 (osteonidogen) | 5.86 | Component of basement membrane, may support cell-matrix adhesion |
| Growth factor independent 1B | 5.86 | Down regulates FOXO1 expression; may promote tumor cell growth/ survival |
| Carbamoyl phosphate synthase 1 | 5.86 | Drives conversion of ammonia to urea in urea cycle; may protect tumor cells from ammonia toxicity during metabolic stress. |
| Caldesmon 1 | 5.66 | Binds actin, myosin and troponin; Inhibits podosome formation and cell motility; supports epithelial organization |
| Inositol 1,4,5 triphosphate receptor type 2 | 5.66 | Interacts with anti-apoptotic protein Bcl-X_L_ to modulate intracellular Ca++ release and induces resistance to apoptosis |
| Adam metallopeptidase domain 12 | 5.66 | Protease activity, releases growth factors; induces signaling through EGFR and IGFR and promotes tumor progression |
| Solute carrier family 26 (sulfate transporter) member 2 | 5.66 | Sulphate transporter, Loss induces abnormal development of chondrocytes, osteoblasts, osteoporosis and bone contraction; supports bone growth |
| Ring finger protein 6 | 5.66 | Ubiquitin E3 ligase activity; Enhances androgen receptor (AR) activity by ubiquitination |
| Ectodysplasin A | 5.66 | TNF family member; induces hormone independent ductal growth via NF-kB |
| KH homology domain containing 1L | 5.66 | Unknown |
| Cadherin 5, type 2 (vascular endothelial) | 5.46 | VEGF mediated signaling receptor; critical to endothelial cell survival |
| Transforming growth factor beta-receptor associated protein 1 | 5.46 | Essential for embryonic development; Binds TGFβ receptor and modulates SMAD-4 activity |
| Zic family member 4 | 5.46 | Transcription factor associated with brain development; expression induced in medulloblastoma |
| Laminin alpha 2 | 5.46 | Component of basement membrane, mediates cell adhesion and inhibits anoikis |
| Acetyl-CoA carboxylase beta | 5.46 | Fatty acid metabolism; cell growth/survival |
| Cyclic nucleotide gated channel , beta 3 | 5.46 | Role in phototransduction in vision |
| Cystein-rich C-terminal 1 | 5.46 | Unknown |
| Glucosamine (Nacetyl)-6-sulfatase | 5.28 | Negatively regulates Wnt signaling; supports epithelial organization |
| N-acylsphingosine amidohydrolase 1 | 5.28 | Catalyzes conversion of proapoptotic ceramide to sphingosine that upon phosphorylation enhances cell growth/survival |
| Inhibin, alpha | 5.28 | INHA serves as a tumor suppressor of gonadal sex cord-stromal cell tumors, however, its role in breast cancer remain unclear |
| Protein tyrosine phosphatase receptor-type alpha | 5.28 | Over expression transforms NIH 3T3 cells; activates Src, FAK and promotes cell adhesion and cell growth/survival |
| Serine/threonine kinase 24 | 5.28 | siRNA treatment induces MCF-7cell migration and reduced E-cadherin expression. supports epithelial organization |
| CDK5 regulatory subunit associated protein 1-like 1 | 5.28 | Loss linked to type 2 diabetes; role in cancer unknown |
| Chromosome 5 open reading frame 4 | 5.28 | Uncharacterized protein |
| Dickkopf homolog 3 | 5.28 | Inhibits T cell immunity |
| PR domain containing 13 | 5.28 | Transcription regulator; role in cancer unknown |
| Carcinoembryonic antigen related cell adhesion molecule 6 | 5.10 | Promotes cell-adhesion; inhibits anoikis; cell growth/survival |
| TSC22 domain family, member 2 | 5.10 | Transcription factor activity; Promotes cell survival in response to osmotic stress |
| Calcium channel, voltage-dependent alpha-2/delta subunit 3 | 5.10 | Expression lost in aggressive neuroblastomas, gastric and breast tumors; putative suppressor of tumor progression |
| 5’ nucleotidase ecto (CD73) | 5.10 | Converts AMP to adenosine; inhibits T cell immunity |
| Activating transcription factor 7 interacting protein | 5.10 | Regulates transcription of telomerase by SP1; ATF7IP overexpression in cultured cells induces telomerase expression; cell growth/survival |
| Family with sequence similarity 198b | 5.10 | Unknown |

Color key for function/role in cancer: Green, cell-cell/cell-matrix adhesion; Pink, regulation of immunity; Dark blue, cell growth/survival; Light blue, bone morphogenesis/ growth and Yellow, transcription regulation. The putative functions for individual genes were assigned based on the literature review.
